# The impact of agricultural technological innovation on residents’ health and its spatial effects

**DOI:** 10.3389/fpubh.2025.1633413

**Published:** 2025-06-25

**Authors:** Yuan Zhao, Shaobo Cui, Yunfeng Xing

**Affiliations:** ^1^Institute of the History of Science and Technology, Inner Mongolia Normal University, Hohhot, Inner Mongolia, China; ^2^School of Economics and Management, Inner Mongolia University of Technology, Hohhot, Inner Mongolia, China; ^3^Baotou Water Conservancy Development Center, Baotou Municipal Water Affairs Bureau, Baotou, Inner Mongolia, China; ^4^School of Agricultural Economics and Rural Development, Renmin University of China, Beijing, China

**Keywords:** agricultural technological innovation, residents’ health, spatial effect, agricultural non-point source pollution, dietary consumption structure

## Abstract

In response to China’s vigorous promotion of health and green, low-carbon development, agricultural technological innovation, as a crucial tool for advancing sustainable development, is essential in promoting residents’ health. Based on data from 30 provinces in China from 2012 to 2022, this paper uses a two-way fixed effects model to examine the impact of agricultural technological innovation on residents’ health. Further, it analyzes the underlying mechanisms through mediation effects and spatial autoregressive models. The research findings are as follows: (1) Agricultural technological innovation can significantly promote residents’ health, with reducing non-point source pollution and improving dietary structure being two important intermediary channels; (2) Agricultural technological innovation has a significant spatial spillover effect on residents’ health, meaning that the health of residents in a region is not only directly influenced by the level of agricultural technological innovation in their region but also indirectly affected by that in neighboring regions.

## 1 Introduction

Health is a vital human capital and a significant support for social and economic development (1). China’s social living standards are constantly improving, but the health conditions of its residents remain worrying. According to the National Health Commission of China, in 2024, the proportion of deaths caused by chronic diseases among total resident deaths exceeded 80%, indicating a severe prevention and control situation for chronic diseases. Moreover, with its high incidence rate, long course of disease, low effective control rate, and heavy economic burden, it has become a significant issue threatening the health of the people and affecting social and economic development. According to the “Report on Nutrition and Chronic Diseases of Chinese Residents (2020),” the incidence rates of hypertension, hypercholesterolemia, chronic obstructive pulmonary disease, diabetes, and cancer have increased compared with the statistics recorded in 2015. To this end, the Chinese government has successively introduced a series of policies aimed at improving the health of residents. For example, in December 2016, the “Healthy China 2030” Plan Outline was released, setting a significant goal to enhance the capacity of health services by 2030. In July 2019, the Healthy China Promotion Committee issued the “Healthy China Initiative (2019–2030).” By April 2025, three actions, namely the healthy weight management action, the healthy rural construction action, and the traditional Chinese medicine health promotion action, were incorporated into the Healthy China Initiative, bringing the total number of special actions to 18. These actions aim to popularize healthy lifestyles and promote the health of the entire population. Against the backdrop of China’s economic development facing an increasingly aging population and the gradual decline of the demographic dividend, the importance of healthy human capital is becoming increasingly prominent.

Previous studies have provided a rich explanation of the factors influencing residents’ health. Some literature focuses on individual and family characteristics at the micro level, finding that residents’ health conditions are affected by factors such as smoking (2), obesity (3), diet (4), income (5), and social capital (6). Another part of the literature focuses on macro external environmental factors, including public services (7), industrial agglomeration (8), and the ecological environment (9). With the in-depth development of agricultural technological innovation, some scholars have begun to recognize that agricultural technological innovation can have a significant impact on health. Wang (10) pointed out that blockchain-enabled agrarian technology has increased the qualification rate of farm products by nearly 30%, which can effectively guarantee the quality and safety of agricultural products. Hansson (11) suggested that agrarian biotechnology can have a positive impact on human health by reducing exposure to pesticides in both occupational and dietary contexts, as well as enhancing the nutritional value of food. Agricultural technological innovation has a positive impact on the quality, safety, and nutritional value of agricultural products. However, how does it specifically affect residents’ health, and what are the underlying mechanisms? These questions have not been explored in existing research.

Given this, this paper empirically analyzes the impact of agricultural technological innovation on residents’ health and its mechanism, based on public data from 30 provinces, using a two-way fixed effects model. This analysis aims to provide theoretical and empirical evidence for improving residents’ health and refining health-related policies. The possible marginal contributions of this paper are as follows: First, it fills the research gap. By focusing on agricultural technological innovation, this study examines whether there is a direct impact on residents’ health, thereby filling the current research gap in the technological perspective on health impacts and enriching the understanding of factors influencing health. Second, it enriches the influence mechanism. This paper integrates agricultural non-point source pollution and dietary consumption structure into the research, examining the underlying mechanism and providing valuable insights into the specific role of agricultural technological innovation in enhancing residents’ health. Third, the empirical analysis using the two-way fixed effects model controls for unobservable individual differences and temporal disturbances, ensuring the consistency and robustness of the results. Meanwhile, it effectively addresses the endogeneity problems caused by provinces and years. Moreover, the spatial spillover effect test of the spatial autoregressive model supplements the regression analysis and demonstrates the cross-spatial impact of agricultural technological innovation.

## 2 Theoretical analysis and research hypotheses

### 2.1 The direct effects of agricultural technological innovation on residents’ health

Agricultural technological innovation can create new knowledge, promote changes in agricultural production methods and innovations in production means, improve agricultural production efficiency (12), and have an undeniable impact on the entire economic and social development. At the same time, it further alters the living and working conditions of residents, and all these changes will affect the health of residents. Agricultural technological innovations, such as biotechnology and precision agriculture technology, are fundamentally transforming agricultural production models (13, 14). The application of these new technologies not only improves the growth conditions of crops, increases yield and quality (15) but also helps ensure the supply capacity of residents’ food and meet their nutritional needs. Moreover, agricultural technological innovation can enhance the degree of automation in production, reduce reliance on physical labor in traditional agriculture (16), and lower labor intensity. This is of great significance for farmers’ physical health, especially in reducing the occurrence of occupational diseases. With the development of digital agricultural technology, the safety and traceability of agricultural products have been significantly improved, thereby enhancing food safety levels. Agricultural technological innovation leads the transformation of agricultural product supply toward nutrition and health (such as selenium-enriched rice), enhances the supply capacity of diversified agricultural products, and thereby improves residents’ health levels. Finally, agricultural technological innovation effectively reduces the use of chemical fertilizers and pesticides, significantly lowering the likelihood of harmful residues in food, thereby ensuring food safety and reducing the risk of public health hazards (17, 18).

Based on this, this paper proposes the following hypotheses:

*Hypothesis* 1: Agricultural technological innovation has a significant positive promoting effect on residents' health.

### 2.2 The mechanism through which agricultural technological innovation influences residents’ health

In recent years, the topic of environmental pollution and its impact on human health has drawn considerable attention. As a typical negative externality, the emergence of environmental pollution has significantly increased health risks to humans (19, 20). Agricultural non-point source pollution is also considered a significant threat to human health. Agricultural non-point source pollution refers to the pollutants generated during agricultural production and rural life, which cause organic, nitrogen, and phosphorus pollution through surface runoff or soil infiltration. It mainly includes various forms of pollution, such as chemical fertilizers and pesticides, livestock and poultry breeding, agricultural films, and solid waste. As a significant source of water and soil pollution, agricultural non-point source pollution not only leads to soil compaction, water eutrophication, and a reduction in biodiversity but also endangers the safety of agricultural product quality (21).

Agricultural technological innovation can effectively reduce non-point source pollution in agriculture (22).the primary ways to achieve this reduction lie in prevention and control. In terms of prevention, precision agriculture technology, biotechnology, and intelligent irrigation can reduce the application of agricultural chemicals (23), improve nutrient utilization efficiency, and enhance crop yield and quality, thereby directly reducing the discharge of agricultural non-point source pollutants from their source. In terms of control, digital technologies, represented by big data, cloud computing, the Internet of Things, and artificial intelligence, empower agricultural technological innovation, providing technical support for monitoring agricultural non-point source pollution, decomposing agricultural waste and pollutants, and controlling and purifying the processes and end products. At the same time, they promote the recycling and reuse of agricultural waste, thereby reducing or eliminating pollutants that enter water bodies and soil and facilitating the control of agricultural non-point source pollution. Reducing agricultural non-point source pollution is essential for ensuring the quality and safety of water and agricultural products, as well as improving the health of residents.

*Hypothesis* 2: Agricultural technological innovation promotes the improvement of residents' health conditions by reducing agricultural non-point source pollution.

In addition, optimizing the dietary consumption structure is another mechanism that mediates the transformation of agricultural technological innovation into improved health conditions for residents. Insufficient dietary energy intake can lead to various health problems, such as anemia, malnutrition, and cardiovascular diseases (24). Optimizing residents’ dietary consumption structure is regarded as an effective way to address this problem (25). Technological innovations, such as facility planting, breeding of superior livestock and poultry varieties, and the development of new aquatic products, have broadened the variety of food supplies, helping residents improve their dietary consumption structure. Agricultural technological innovations, such as superior variety breeding, mechanization, water-saving irrigation, and pest and disease control, have increased the yield per unit area of grains, vegetables, fruits, and livestock and poultry products (26), making the nutrients that residents consume more comprehensive, including protein, vitamins, and trace elements. Improving the dietary consumption structure is conducive to enhancing immunity and reducing the risk of related diseases, thus having a relatively significant positive impact on residents’ health.

*Hypothesis* 3: Agricultural technological innovation promotes the improvement of residents' health conditions by enhancing the dietary consumption structure.

### 2.3 The spatial spillover effects of agricultural technological innovation on residents’ health

The impact of agricultural technological innovation on residents’ health may have a spatial effect. First, agricultural technological innovation increases the output, quality, and variety of agricultural products. The agricultural products produced in this region can be transported to other places for sale through logistics. Residents in other regions can obtain more nutritious and safer food, thus improving their health levels. Second, agricultural production exhibits a regional characteristic of being concentrated in contiguous areas, which means that agricultural production between regions often shows spatial correlation. The technological changes resulting from agricultural innovation activities will produce apparent spatial spillover effects, leading to the fact that local agricultural technological innovation will affect the health of residents in neighboring regions through these spatial spillovers. Third, agricultural non-point source pollution is carried by water. Soluble or solid agricultural pollutants, under the action of precipitation or irrigation, will flow into water bodies through surface runoff, farmland drainage, and underground seepage. This not only causes local water environmental pollution but also easily damages the surrounding environment with similar water systems and topography (27), thereby affecting the health level of residents in surrounding regions.

*Hypothesis* 4: The promoting effect of agricultural technological innovation on residents' health has a spatial spillover effect.

## 3 Model specification, variables and data sources

### 3.1 Model specification

#### 3.1.1 Benchmark model

To examine the relationship between agricultural technological innovation and residents’ health, this paper adopts a two-way fixed effects model (28), which is set up as follows:


(1) $$\;H_{\mathrm{it}}=\beta_{0}+\beta_{1}{\mathrm{ATI}}_{\mathrm{it}}+\beta X_{\mathrm{it}}+\mu_{i}+\delta_{t}+\varepsilon_{\mathrm{it}}$$

In Equation 1, H_it_ represents the health level of residents in province I at period t, ATI_it_ represents the level of agricultural technological innovation in province I at period t, and X represents control variables; β_0_ is the constant term, μ_i_ and δ_t_ represents the fixed effects of provinces and years respectively, and ε_it_ represents the random error term.

#### 3.1.2 Mediation effect model

To verify whether agricultural technological innovation promotes the improvement of residents’ health levels through two mediating channels of reducing non-point source pollution and improving dietary consumption structure, the following mediation effect model is constructed (29):


(2) $$\;{\mathrm{Med}}_{\mathrm{it}}=\gamma_{0}+\gamma_{1}{\mathrm{ATI}}_{\mathrm{it}}+\delta_{0}X_{\mathrm{it}}+\mu_{i}+\delta_{t}+\varepsilon_{\mathrm{it}}$$

(3) $$\;H_{\mathrm{it}}=\theta_{0}+\theta_{1}{\mathrm{ATI}}_{\mathrm{it}}+\theta_{2}{\mathrm{Med}}_{\mathrm{it}}+\varphi_{0}X_{\mathrm{it}}+\mu_{i}+\delta_{t}+\varepsilon_{\mathrm{it}}$$

In Equations 2, 3, Med_it_ represents the mediating variables, including two types of mediating variables: reducing non-point source pollution and improving dietary consumption structure. The definitions of the remaining variables are consistent with Equation 1.

#### 3.1.3 Spatial correlation test

The Global Moran’s I is selected to conduct the Moran test (30). The specific formula is as follows:


$$\text{Global Moran}\prime s\;I=\frac{N}{S}\frac{\sum_{i=1}^{N}\sum_{j=1}^{N}W_{\mathrm{ij}}Y_{i}Y_{j}}{\sum_{i=1}^{N}Y_{i}^{2}}$$

Among them, W_ij_ represents the spatial weight between provinces, and an adjacency spatial weight matrix is selected. X_i_ successively represents the development level of the agricultural production service industry and the rural industrial revitalization index of each province in China. Y_i_ = X_i_ - $\bar{X}$ is the difference between the value of province i and the average value of all provinces. N is the number of provinces, and S is the aggregation of spatial weights between all provinces. The setting of the adjacency spatial weight matrix is as follows:


$$W_{\mathrm{ij}}=\{\begin{array}{c} 1,\text{The spaces of}\;i\;\text{and}\;j\;\mathrm{are}\;\text{adjacent}.\\ \;0,\text{The spaces of}\;i\;\text{and}\;j\;\mathrm{are}\;\text{not adjacent}. \end{array}$$

#### 3.1.4 Spatial econometric model

Following Lin’s approach (31), the Spatial Autoregressive Model (SAR) is selected to analyze the spatial effect of agricultural technological innovation on residents’ health, and the following model is constructed:


(4) $$\;H_{\mathrm{it}}=\alpha_{0}+\mathrm{\rho W}H_{\mathrm{it}}+\alpha_{1}{\mathrm{ATI}}_{\mathrm{it}}+\alpha X_{\mathrm{it}}+\mu_{i}+\delta_{t}+\varepsilon_{\mathrm{it}}$$

In Equation 4: ρ is the spatial autocorrelation coefficient, $\alpha_{0}$ is a constant term, W is the spatial weight matrix, and $\;\alpha_{1}$ is the coefficient of the core explanatory variable; α is the parameter to be estimated. The remaining variables and coefficients are set consistently with Equation 1.

### 3.2 Variable selection and explanation

#### 3.2.1 Explained variable: residents’ health

With the development of micro-databases and questionnaires in recent years, “self-rated health” has been widely regarded by scholars as a key indicator of health status due to its ability to represent an individual’s overall health condition. However, given that self-rated health indicators are subject to a certain degree of personal subjective factors, for the sake of caution, this paper will adopt objective health indicators to examine the health status of Chinese residents comprehensively. Drawing on existing research results (32–34), an evaluation index system for residents’ health levels is constructed, which includes two first-level indicators and seven second-level indicators. The entropy value method is used to calculate the residents’ health level comprehensively, and this value serves as a proxy variable for measuring residents’ health, as shown in Table 1.

**Table 1:** Evaluation index system for residents’ health status.

Primary indicators	Secondary indicators	Indicator attributes	Indicator weights
Overall health level	The incidence rate of Class A and B notifiable infectious diseases	−	0.178
	Average life expectancy of the population	+	0.350
	Medical system management rate	+	0.085
The health level of pregnant women and children	The rate of hospital deliveries	+	0.032
	Perinatal infant mortality rate	−	0.088
	The management rate of children under 3 years old in the system	+	0.071
	The prevalence of low weight among children under 5 years old	−	0.196

#### 3.2.2 Core explanatory variable: agricultural technological innovation

Based on the principles of data availability, indicator scientificity, comprehensiveness, and hierarchy, and drawing on the research of Gao (35) and Wang (36), this study adopts three dimensions of agricultural technological innovation: information technology, production efficiency, and circulation efficiency. The level of agricultural technological innovation is comprehensively evaluated through the entropy method and used as a proxy variable for agricultural technological innovation, as detailed in Table 2. Information technology serves as the foundation of agricultural technological innovation, measuring the extent of its popularization in innovation and reflecting the “intellectual support” and infrastructure capacity of agricultural technological innovation. Production efficiency focuses on the performance of agricultural technological innovation in enhancing the output per unit of resources, such as output per unit area, rural electricity levels, and agricultural mechanization levels. It reflects the ability of fundamental agricultural innovation to replace and optimize traditional production methods. Circulation efficiency reflects the smoothness of logistics in rural areas and is a crucial indicator of the circulation and dissemination of agricultural technological innovations.

**Table 2:** Evaluation index system of agricultural technological innovation level.

Primary index	Secondary index	Index explanation	Index attribute	Index weight
Information Technology	The penetration rate of mobile phones	The average number of mobile phones owned by rural households per 100 households at the end of the year	+	0.026
	The popularization of computers	The average number of computers owned by rural households per 100 at the end of the year	+	0.058
	Meteorological stations use	The number of agricultural meteorological observation business stations	+	0.068
production efficiency	output value per unit area	Total agricultural output value / total sown area of crops	+	0.125
	The level of rural electricity supply	Rural electricity consumption / rural population	+	0.467
	The level of agricultural mechanization	Total power of agricultural machinery / Total sown area of crops	+	0.119
Circulation efficiency	Rural delivery routes	Rural delivery route kilometers	+	0.094
	Rural delivery frequency	Average number of weekly deliveries in rural areas	+	0.039
	Postal service level	The proportion of administrative villages with postal services	+	0.004

#### 3.2.3 Mediating variables: agricultural non-point source pollution emission and dietary consumption structure

Due to the characteristic of being difficult to monitor, the discharge of agricultural non-point source pollution is hard to calculate statistically, and the estimation methods are not unified. Among them, the unit investigation method ensures the comprehensiveness, typicality, and representativeness of the investigation units and also fully considers the availability, comparability, and significance of the investigation statistical data. Therefore, it is widely used in the current assessment of agricultural non-point source pollution. Thus, this paper adopts this method to calculate the discharge of agricultural non-point source pollution.

First, identifying pollution categories and analyzing pollution generation. The harmful substances of agricultural non-point source pollution include chemical oxygen demand (COD), total nitrogen (TN), and total phosphorus (TP). Considering that COD is difficult to estimate using literature data, the pollutants of focus in this study are total nitrogen (TN) and total phosphorus (TP) (27).secondly, the determination of pollution generation units. Pollution generation units refer to non-point source pollution sources, which are independent units that directly generate pollutants and contribute a certain percentage to non-point source pollution. Agricultural non-point source pollution occurs during agricultural production and rural life. Fertilizers are a significant source of pollution in agricultural production. Since the leading causes of environmental pollution in fertilizers are nitrogen and phosphorus fertilizers, and potassium fertilizers do not directly cause non-point source pollution, the pollution generation units in agricultural production can be identified as nitrogen fertilizers, phosphorus fertilizers, and compound fertilizers.

Regarding rural life, rural domestic sewage is considered the primary source of pollution generation. Thirdly, the determination of unit emission coefficients. The emission coefficient is equal to the pollution generation coefficient multiplied by the fertilizer loss rate. For the pollution generation coefficients of each unit, this study follows the standard practice in previous literature (37). It calculates them based on the chemical composition of the pure fertilizer: the TN pollution generation coefficients of nitrogen fertilizers, phosphorus fertilizers, and compound fertilizers are 1, 0, and 0.33, respectively, and the TP pollution generation coefficients of nitrogen fertilizers, phosphorus fertilizers, and compound fertilizers are 0, 0.44, and 0.15, respectively. The loss intensities of TN and TP in the rural domestic sewage unit are 0.89 and 0.2, respectively (38).finally, assessment of agricultural non-point source pollution. Based on the unit analysis results, the total number of units, pollution generation coefficients, and loss rates can be obtained. Based on this, the emission of agricultural non-point source pollutants can be further estimated, as shown in Table 3.

**Table 3:** Determination of accounting units for agricultural non-point source pollution.

Pollution source	Pollution unit	Measurement method
Application of chemical fertilizers	Nitrogen fertilizer, phosphorus fertilizer, compound fertilizer	Total nitrogen emissions = (Pure nitrogen amount of nitrogen fertilizer + Pure nitrogen amount of compound fertilizer × 0.33) × Nitrogen loss coefficient Total phosphorus emissions = (phosphate fertilizer equivalent × 0.44 + compound fertilizer equivalent × 0.15) × phosphorus loss coefficient
Rural life	Rural domestic sewage	Total nitrogen emissions = Rural population × Nitrogen loss intensity (kg/person) Total phosphorus emissions = Rural population × Phosphorus loss intensity (kg/person)

Optimizing dietary structure refers to the proportion of various food types in the diet. The main factors influencing the dietary and nutritional status of residents include grains, edible oils, meat, eggs, milk, and aquatic products. According to the statistical standards of the “China Statistical Yearbook,” grains include cereals, tubers, and legumes; animal source foods include meat (pork/beef/mutton, poultry), eggs, milk, and aquatic products. This paper focuses on analyzing the consumption characteristics of per capita vegetables, meat, poultry, and aquatic products of residents. It incorporates these dietary consumption structures as mechanism analysis variables into the research to clarify the influence mechanism of agricultural technological innovation on the health status of residents.

#### 3.2.4 Control variables

To eliminate the influence of other factors on residents’ health, referring to relevant studies (39, 40), the control variables are selected as air pollution, aging level, education level, fiscal revenue-to-expenditure ratio, industrial structure, medical coverage, and social security coverage. Air pollution is represented by the sulfur dioxide emissions (in 10,000 tons) of each province; the aging level is indicated by the proportion of the population aged 65 and above (%); the education level is measured by the average years of education per capita in the region; the fiscal revenue-to-expenditure ratio is characterized by the general public service expenditure of local finance / general budget revenue of local finance (%); the industrial structure is expressed by the added value of the primary industry / (added value of the primary industry + added value of the secondary industry + added value of the tertiary industry; %); medical coverage is measured by the number of hospital beds (in 10,000) / the permanent resident population at the end of the year (per person); social security coverage is represented by the number of participants in the social endowment insurance for urban and rural residents / the permanent resident population at the end of the year (%). The descriptive statistics of each variable are shown in Table 4.

**Table 4:** Descriptive statistical results of variables.

Variable types	Variable name	Indicator calculation	Obs	Mean	Std. dev.	Min	Max
The explained variable	Residents’ health level	Obtained through calculation	341	0.704	0.126	0.125	0.961
Key explanatory variable	Agricultural technological innovation	Obtained through calculation	341	0.187	0.072	0.077	0.589
mediating variable	Agricultural non-point source pollution	Obtained through calculation	341	18.708	15.524	0.523	65.91
	Dietary consumption structure	Obtained through calculation	341	0.323	0.177	0.049	0.868
Control variable	Air pollution	Sulfur dioxide emissions	341	33.052	35.348	0.11	174.88
	The level of aging	The proportion of the population aged 65 and above	341	0.112	0.03	0.05	0.2
	Educational level	Average years of education per capita	341	9.206	1.129	4.22	12.78
	The ratio of fiscal revenue to expenditure	The proportion of local fiscal public service expenditure to revenue	341	0.251	0.243	0.047	1.9
	industrial structure	The proportion of the added value of the primary industry	341	0.097	0.052	0.002	0.253
	Medical coverage	Number of hospital beds per 10,000 people	341	0.004	0.001	0.002	0.007
	Social security coverage	Participation rate of the urban and rural residents’ endowment insurance	341	0.353	0.136	0.029	0.575

### 3.3 Data sources

This study selected panel data from 2012 to 2022 of 30 provincial regions (excluding Hong Kong, Macao, Taiwan, and Tibet regions), which were, respectively, from the China Statistical Yearbook, China Rural Statistical Yearbook, China Health Statistical Yearbook, China Tertiary Industry Statistical Yearbook, and provincial statistical yearbooks and the National Bureau of Statistics. In addition, data from the EPS database and DRCnet database were also utilized. To address the data missing problem in some regions, this paper employs the interpolation method to complete the data, thereby ensuring the integrity and accuracy of the analysis.

## 4 Empirical results analysis

### 4.1 Benchmark regression

Table 5 reports the benchmark regression results. Column (1) and column (2) respectively present the mixed OLS regression without control variables and the two-way fixed effects model estimation without control variables. Column (3) and column (4) report the two-way fixed effects model estimation with only air pollution and aging level variables included and with all variables included. The regression results indicate that the core explanatory variables are significantly positive at the 1% level, suggesting that agricultural technological innovation has a substantial positive impact on residents’ health levels. The reason for this is that agricultural technological innovation enriches the variety of agricultural products, promotes the upgrading of dietary consumption structure, and promotes the rationalization and scientification of rural residents’ dietary intake, shifting from “eating enough” to “eating healthily,” thereby improving residents’ health levels. In addition, agricultural technological innovation can reduce the application of agricultural chemicals, improve nutrient utilization efficiency, and reduce the discharge of agricultural non-point source pollutants, which is conducive to ensuring the safety of water and soil environments and helps to improve residents’ health conditions. Therefore, Hypothesis 1 is verified.

**Table 5:** Benchmark regression results.

Variable	(1) OLS	(2) FE	(3) FE	(4) FE
Agricultural technological innovation	0.796*** (9.487)	0.125*** (4.114)	0.096*** (4.945)	0.084*** (4.744)
Air pollution			0.001** (1.978)	0.001* (1.733)
The level of aging			−0.940*** (−4.233)	−0.826*** (−3.877)
Educational level				0.005 (0.468)
The ratio of fiscal revenue to expenditure				−0.109** (−1.992)
industrial structure				−0.306 (−1.519)
Medical coverage				11.651 (1.640)
Social security coverage				0.246*** (3.085)
province	N	Y	Y	Y
year	N	Y	Y	Y
Constant term	0.555*** (32.937)	0.680*** (120.426)	0.785*** (30.945)	0.649*** (5.479)
N	341	341	341	341
R^2^	0.210	0.967	0.971	0.975

The figures in parentheses are standard errors; ***, **, and * indicate significance at the 1, 5, and 10% levels, respectively.

Among the control variables, air pollution shows a significant negative impact in columns (3) and (4), being significant at the 5 and 10% levels, respectively. This indicates that the decline in environmental quality may have a restraining effect on residents’ health. Notably, the aging level is negative and highly significant in all fixed-effects models, suggesting that the increase in the degree of population aging significantly restrains the health level of residents. This reflects that aging not only directly affects the health of the older adults through physiological health burdens but also impacts the health of all residents through systemic shocks to medical resources, fiscal capacity, and social structure. The fiscal revenue-to-expenditure ratio is significantly negative at the 5% level, which may indicate that the tight fiscal state restricts related expenditures or investment capabilities, thereby inhibiting the improvement of residents’ health levels. Social security coverage, on the other hand, shows a significant positive effect, indicating that a sound social security system helps to enhance residents’ health levels. Other control variables, such as education level, industrial structure, and medical coverage, do not show significant effects in the models, which may be due to multicollinearity or overlapping explanatory power with the included variables. From the perspective of model fit, the R^2^ value rapidly increases from 0.210 in the OLS model to 0.975 in the fixed-effects model, indicating that the introduction of provincial and year-fixed effects has significantly enhanced the model’s explanatory power.

### 4.2 Mechanism analysis

The results of the mechanism test are presented in Table 6. The findings reveal that agricultural technological innovation significantly reduces agricultural non-point source pollution (*β* = −2.841, *p* < 0.1), indicating that advancements in agrarian technology contribute to the reduction of agricultural pollutant emissions such as fertilizers and pesticides, optimize agrarian production methods, and indirectly improve the quality of the ecological environment, thus forming an important path for promoting the improvement of residents’ health. Agricultural technological innovation has a significant positive impact on dietary consumption structure (*β* = 0.080, *p* < 0.1), suggesting that agrarian innovation may guide residents toward a more scientific and reasonable dietary structure by enhancing the diversity of agricultural products and improving the capacity for nutritional supply, thereby promoting the improvement of residents’ health levels. Therefore, Hypotheses 2 and 3 are supported.

**Table 6:** Results of mechanism analysis.

Variable	(1) Agricultural non-point source pollution	(2) Dietary consumption structure
Agricultural technological innovation	−2.841* (−1.677)	0.080* (1.807)
Control variable	Under control	Under control
province	Y	Y
year	Y	Y
Constant term	11.337** (2.147)	0.571*** (5.098)
N	341	341
R^2^	0.992	0.981

The figures in parentheses are standard errors; ***, **, and * indicate significance at the 1, 5, and 10% levels, respectively.

### 4.3 Analysis of spatial spillover effects

Table 7 reports the global Moran’s Index of agricultural technological innovation and residents’ health from 2012 to 2022. It can be observed that the global Moran’s Index for both variables is significantly positive, indicating a significant spatial autocorrelation between agricultural technological innovation and residents’ health. They show a certain degree of positive agglomeration characteristics in spatial distribution. This suggests that a spatial econometric model can be used for empirical analysis.

**Table 7:** Global Moran’s index of agricultural technological innovation and residents’ health.

Year	Agricultural technological innovation			Residents’ health level		
	Moran’s I	*z* value	*p* value	Moran’s I	*z* value	*p* value
2012	0.211	2.766	0.003	0.278	3.779	0
2013	0.256	3.834	0	0.299	3.93	0
2014	0.27	3.972	0	0.31	4.077	0
2015	0.259	3.8	0	0.308	4.075	0
2016	0.261	3.992	0	0.345	4.427	0
2017	0.24	3.722	0	0.345	4.411	0
2018	0.223	3.51	0	0.344	4.41	0
2019	0.237	3.688	0	0.331	4.328	0
2020	−0.015	0.208	0.417	0.361	4.636	0
2021	−0.005	0.325	0.372	0.367	4.718	0
2022	−0.001	0.369	0.356	0.364	4.667	0

Table 8 presents the SAR test results. The spatial autoregressive coefficient of agricultural technological innovation in neighboring provinces is significantly positive and passes the 1% significance level. This indicates that there is mutual influence among different individuals of agricultural technological innovation in terms of space. The evaluation of independent variables in the spatial econometric model is a crucial aspect of spatial econometric analysis, encompassing the assessment of direct, indirect, and total effects. As shown in the table, the direct effect of agricultural technological innovation on residents’ health is statistically significant at the 1% level, and the indirect effect is statistically significant at the 5% level. This shows that agricultural technological innovation has a positive spatial spillover effect on the health of residents in the local area and neighboring regions, thereby confirming Hypothesis 4.

**Table 8:** Test results of spatial autoregressive model.

Variables	(1)	(2)	(3)	(4)	(5)	(6)
	Main	Spatial	Variance	Direct	Indirect	Total
ATI	0.090*** (2.811)			0.094*** (2.812)	0.081** (2.244)	0.176*** (2.656)
Air	0.000 (1.629)			0.001* (1.794)	0.001 (1.529)	0.001* (1.692)
Old	−0.158 (−1.203)			−0.157 (−1.047)	−0.140 (−1.013)	−0.297 (−1.041)
Edu	0.031*** (4.745)			0.033*** (4.557)	0.028*** (3.780)	0.061*** (4.763)
Fin	−0.083*** (−3.125)			−0.093*** (−2.884)	−0.079** (−2.386)	−0.172*** (−2.789)
Ind	−0.378** (−2.344)			−0.382** (−2.308)	−0.335* (−1.878)	−0.717** (−2.138)
Med	19.339*** (4.362)			19.944***(4.016)	16.839*** (3.327)	36.783*** (4.046)
Sec	0.266*** (4.259)			0.270*** (4.401)	0.233*** (2.905)	0.503*** (3.808)
rho	0.482*** (6.600)					
sigma^2^	0.001*** (12.851)					
N	341	341	341	341	341	341
R^2^	0.612	0.612	0.612	0.612	0.612	0.612

The figures in parentheses are standard errors; ***, **, and * indicate significance at the 1, 5, and 10% levels, respectively.

## 5 Discussion

This study uses data from the China Statistical Yearbook, China Rural Statistical Yearbook, China Tertiary Industry Statistical Yearbook, and the National Bureau of Statistics to construct a comprehensive measurement index for agricultural technological innovation from three dimensions: Information Technology, production efficiency, and Circulation efficiency. The level of residents’ health is measured using relevant data from the China Health Statistics Yearbook and National Public Service Statistics Yearbook. On this basis, the two-way fixed effects model and spatial autoregressive model were used to empirically analyze the impact mechanism of agricultural technological innovation on residents’ health and identify its spatial spillover effects. The following are three valuable findings:

Firstly, this study demonstrates that agricultural technological innovation has a positive impact on the health level of residents. The possible reasons are that agricultural technological innovation adopts green technologies, such as efficient irrigation and precise fertilization, to ensure that crops obtain the optimal amount of nutrients they need and avoid excessive application of chemical fertilizers and pesticides (41, 42). The development of genetically modified crops resistant to pests and diseases, along with biological control technologies, has enhanced the disease resistance of crops, reduced their reliance on pesticides, significantly improved agricultural production efficiency, ensured the quantity and quality of food, and thus promoted improvements in residents’ health levels. This result further expands the research of Xu (21), who believes that the technological progress of agricultural machinery is positively correlated with residents’ health. Our research shows that current agricultural technological innovation not only focuses on improving agricultural production efficiency but also emphasizes its role in protecting the ecological environment and promoting sustainable development. Agricultural technological innovation is undergoing a green transformation, aiming to promote a healthier and more sustainable agricultural production model. Overall, agricultural technological innovation can also enhance the health level of residents.

Secondly, this study examines the mechanism by which agricultural technological innovation impacts residents’ health. The research finds that the main pathways include: Firstly, by reducing agricultural non-point source pollution, such as precision fertilization technology, which changes the amount and mixture of fertilizers and pesticides based on field needs to improve their application, thereby achieving precise management through enhanced resource utilization (43), reducing the intensity of chemical fertilizer and pesticide application and lowering the health burden on residents exposed to environmental risks. Additionally, technologies such as nitrogen reduction in fertilizers, partial substitution of chemical fertilizers with organic fertilizers, and biological nitrogen fixation can all achieve the effect of reducing chemical fertilizer application while increasing efficiency and reducing environmental pollution. This conclusion further validates the theoretical judgment made by Qin Tian (44) scholars that there is a significant negative correlation between agricultural non-point source pollution and citizens’ health levels. Second, optimizing the dietary structure enhances the possibility for residents to obtain rich and nutritious food, thereby improving their health conditions. Technologies such as genetic modification and agricultural breeding can cultivate crop varieties that are more productive, disease-resistant, and resilient to adverse conditions, thus enriching the variety of farm products and providing residents with a more diverse food supply. Innovative greenhouse technology not only increases crop yields, extends the production cycle, and improves crop quality but also enriches crop varieties and enhances the diversity of food supply. By maintaining the quality and nutritional content of food through temperature and humidity monitoring, as well as environmental control technologies, in the agricultural supply chain, consumers can access a greater variety of fresh food. These technological innovations enable residents to access a more diverse range of food, thereby improving their dietary structure and promoting an increase in health levels (45). This is consistent with Sheng’s (46) research conclusion, which holds that adopting a reasonable dietary structure will significantly reduce the incidence and mortality rates of diet-related chronic diseases.

Thirdly, it further reveals the spatial spillover effect of agricultural technological innovation on residents’ health. In previous studies, the spatial effects of technological innovation have been widely emphasized (47). Cai (48), Tian (49), and Feng (50) have studied the spatial spillover effects of technological innovation on new urbanization, carbon neutrality, and carbon emissions. Our research further expands the scope of the spatial effect of technological innovation. Agricultural technological innovation may also have spatial spillover effects on residents’ health through multiple aspects, such as information dissemination, technology diffusion, environmental improvement, and market circulation, which can affect the quality of life and health of residents in the broader area. Our research results are consistent with the work of Wang (51) and extend their work. They found that the development of agricultural science and technology innovation in China exhibits an obvious spatial correlation and spillover effect, while this study extends the spatial spillover of agricultural technological innovation to its impact on residents’ health, thereby revealing its broader social benefits.

## 6 Conclusion and implications

This study explores the influence mechanism and spatial effects of agricultural technological innovation on residents’ health. The following conclusions are drawn: (1) Agricultural technological innovation can effectively enhance residents’ health by reducing non-point source pollution and improving dietary consumption structure. (2) It is confirmed that agricultural technological innovation exhibits significant spatial spillover effects in terms of residents’ health. Based on the empirical findings of this study, the following policy recommendations are proposed to more effectively leverage agricultural technological innovation in enhancing residents’ health.

Firstly, the government should increase its support for the research and development of green agricultural technologies, particularly in key areas such as reducing the use of chemical fertilizers and pesticides, enhancing resource utilization efficiency, and promoting ecological agriculture. To encourage agricultural operators to adopt efficient, low-pollution, and health-promoting production technologies, the government should utilize a diversified range of measures, including financial subsidies, technical guidance, and talent development, to motivate farmers and agricultural enterprises to adopt these innovative technologies actively.

Secondly, efforts should be made to promote the cross-regional diffusion and sharing of agricultural technological innovation achievements. The government should increase financial support for the research, development, application, and promotion of green agricultural technologies, with particular attention given to the technological penetration in regions with high resource-carrying capacity and high population density. Given that agricultural technologies have significant spatial spillover effects, it is recommended that a regional agricultural science and technology collaboration mechanism be established. This can be achieved through technology exchange platforms, pilot demonstration projects, and cross-provincial and regional joint research programs to enhance the penetration and accessibility of technologies in less developed areas, thereby achieving a healthy and fair collaborative improvement among regions.

Thirdly, in the formulation of agricultural science and technology policies, greater emphasis should be placed on integrating the goal of “health orientation.” While enhancing agricultural productivity, the health of residents should not be compromised. Policies that encourage green agricultural technological innovation should be continuously advanced, and health benefit evaluation indicators should be introduced into relevant policy tools to enhance consideration of health externalities. Additionally, the agricultural sector should strengthen collaborative governance with public health, ecological environment, and other relevant departments and establish a cross-departmental linkage mechanism to promote the construction of healthy agriculture from a systemic perspective.

Finally, efforts should be made to promote the establishment and improvement of a dynamic monitoring system for agricultural technological innovation and residents’ health conditions. For instance, conducting long-term tracking of the application of genetically modified agricultural technologies and residents’ health data to form a data loop will help to assess the actual effectiveness of technological innovation more scientifically and provide data support for policy adjustments and technological optimization.

## Data Availability

Publicly available datasets were analyzed in this study. This data can be found at: the raw data supporting the conclusions of this article will be made available on request.
